# What does the future of quality improvement look like?

**DOI:** 10.1093/intqhc/mzae070

**Published:** 2024-07-13

**Authors:** Amar Shah, Rosa Sunol

**Affiliations:** East London NHS Foundation Trust, London, UK; NHS England, UK; Royal College of Psychiatrists, London, UK; Avedis Donabedian Research Institute (FAD), Barcelona, Spain; Universidad Autònoma de Barcelona, Barcelona, Spain; Network for Research on Chronicity, Primary Care, and Health Promotion (RICAPPS), Spain

Quality improvement is the application of a systematic method to work through complex problems, involving the people closest to the issue in discovering new solutions, testing the ideas in a structured way, and learning through the use of data [1]. There are excellent examples of what can be achieved, where this approach has been adopted, embedded and sustained over many years [2, 3]. This includes both organizational journeys, as well as demonstrations of applying the method at scale across multiple organizations in order to tackle common challenges [4].

There is often, though, inadequate rigour in the way we apply quality improvement, with the consequence of results not being achieved and a lack of belief in this approach to problem-solving [5]. Truly adopting this way of working takes time, persistence, and constancy of purpose. There is growing evidence about the key components needed to adopt and embed quality improvement in organizations and systems. Four key aspects emerge—first, an ongoing effort to build and deepen capacity and capability for improvement [6]. Second, careful nurturing and development of a culture and context that provides staff and service users with the autonomy, skills, and permission to test creative ideas. Third, leadership behaviours that create psychological safety and reinforce problem-solving at the point of care, supported by policy initiatives and incentives for high quality. And fourth, the development of a robust infrastructure to ensure that teams have close, skilled support as they tackle some of the most complex challenges in the organization [7].

Building improvement capability enables organizations to call on the experience and expertise of skilled improvers who can support robust and rigorous design for large-scale improvement and implementation, enabling the organization to tackle strategic issues at scale. This includes the use of a range of designs, which are hitherto relatively underutilized in healthcare, such as factorial experimental design, and the use of evaluation, both formative and summative, in order to learn and adapt [8].

A key stumbling block has often been the ability to scale and spread improvement. In an industry that is perhaps more complex than any other, this needs careful and expert planning. Often, scale and spread efforts have failed because the original demonstrator site has failed to convincingly show improvement, the set of change ideas has not been tested adequately in a range of different contexts, or because the design and infrastructure for scale-up have been inadequate. There is an opportunity here to harness large-scale change approaches, and the learning from implementation science, to enable the more effective adoption and spread in healthcare [9].

The future of quality improvement will be influenced by the advent of generative artificial intelligence (AI). The ability for teams to rapidly harness existing knowledge in building a theory, learning from previous similar improvement efforts, synthesizing qualitative or quantitative data, could accelerate quality improvement, so that more precious clinical time is spent on actually making change happen. This has the potential to alleviate one of the main barriers to quality improvement, which is time to do the work. However, the risks and hazards with generative AI, including data security and bias, still need further evaluation and mitigation.

If the approach of quality improvement is truly to be adopted at scale across healthcare systems, we need to demonstrate that it can deliver results in all domains of quality, including efficiency, productivity, sustainability, and equity. The evidence in some of these areas is still nascent but growing. Some of the challenges we currently face are most likely to require an approach that encourages experimentation and utilizes the creativity and wisdom of both the workforce and those with lived experience. To harness this effectively, we need to ensure greater rigour in our application, with sufficient capacity and capability, leadership behaviours that encourage testing and learning, and the development of close, skilled support for teams as they work through complex problems.

## References

[1] Shah A . How to move beyond quality improvement projects. *BMJ* 2020;370:m2319.doi: 10.1136/bmj.m2319 32718988

[2] Fostering an Improvement Culture: Learning from East London NHS Foundation Trust’s Improvement Journey Over 10 Years. Boston: Institute for Healthcare Improvement, 2024. https://www.ihi.org/resources/publications/fostering-improvement-culture-learning-east-london-nhs-foundation-trusts.

[3] Burgess N, Currie G, Crump B et al. Leading change across a healthcare system: how to build improvement capability and foster a culture of continuous improvement. Report of the Evaluation of the NHS-VMI partnership. Warwick Business School, 2022.

[4] Shah A, Akhtar S et al. Increasing joy in work in UK healthcare teams: a national quality improvement collaborative. *Br J Healthc Manage* 2023;29:1–16.doi: 10.12968/bjhc.2022.0139

[5] Shah A . The rigour of quality improvement work – why it matters, and what it looks like. *BMJ Open Quality* 2023;12. doi: 10.1136/bmjoq-2023-ISS.1

[6] Frasquilho F, Brittin K et al. Building capacity and capability for quality improvement: developing an organisational approach. *Br J Healthc Manage* 2023;29:1–14.doi: 10.12968/bjhc.2023.0003

[7] Kaplan HC, Provost LP, Froehle CM et al. The Model for Understanding Success in Quality (MUSIQ): building a theory of context in healthcare quality improvement. *BMJ Qual Saf* 2012;21:13–20.doi: 10.1136/bmjqs-2011-000010 21835762

[8] Dixon-Woods M, McNicol S, Martin G. Ten challenges in improving quality in healthcare: lessons from the Health Foundation’s programme evaluations and relevant literature. *BMJ Qual Saf* 2012;21:876–84.doi: 10.1136/bmjqs-2011-000760 PMC3461644 22543475

[9] Greenhalgh T, Papoutsi C. Spreading and scaling up innovation and improvement. *BMJ* 2019;365:l2068.doi: 10.1136/bmj.l2068 PMC6519511 31076440

